# Comparative transcriptomics from intestinal cells of permissive and non-permissive hosts during *Ancylostoma ceylanicum* infection reveals unique signatures of protection and host specificity

**DOI:** 10.1017/S0031182023000227

**Published:** 2023-05

**Authors:** Andrea Langeland, Emilia Grill, Amol C. Shetty, Damien M. O'Halloran, John M. Hawdon

**Affiliations:** 1 Department of Biological Sciences, The George Washington University, Washington DC, USA; 2 Department of Microbiology, Immunology, and Tropical Medicine, The George Washington University, Washington DC, USA; 3 Institue for Genome Sciences, University of Maryland School of Medicine, Baltimore, Maryland, USA

**Keywords:** *Ancylostoma ceylanicum*, differential expression, hookworm, host specificity, rodents

## Abstract

Soil-transmitted nematodes (STNs) place a tremendous burden on health and economics worldwide with an estimate of at least 1.5 billion people, or 24% of the population, being infected with at least 1 STN globally. Children and pregnant women carry the heavier pathological burden, and disease caused by the blood-feeding worm in the intestine can result in anaemia and delays in physical and intellectual development. These parasites are capable of infecting and reproducing in various host species, but what determines host specificity remains unanswered. Identifying the molecular determinants of host specificity would provide a crucial breakthrough towards understanding the biology of parasitism and could provide attractive targets for intervention. To investigate specificity mechanisms, members of the hookworm genus *Ancylostoma* provide a powerful system as they range from strict specialists to generalists. Using transcriptomics, differentially expressed genes (DEGs) in permissive (hamster) and non-permissive (mouse) hosts at different early time points during infection with *A. ceylanicum* were examined. Analysis of the data has identified unique immune responses in mice, as well as potential permissive signals in hamsters. Specifically, immune pathways associated with resistance to infection are upregulated in the non-permissive host, providing a possible protection mechanism that is absent in the permissive host. Furthermore, unique signatures of host specificity that may inform the parasite that it has invaded a permissive host were identified. These data provide novel insight into the tissue-specific gene expression differences between permissive and non-permissive hosts in response to hookworm infection.

## Graphical abstract

**Figure:**
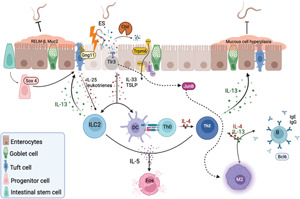

## Introduction

Hookworm infection is a devastating disease affecting approximately 500 million people worldwide, and is particularly harmful in children and pregnant women (Pullan *et al*., 2014). Disease caused by the blood-feeding worm in the intestine can lead to anaemia as well as deylas in physical and intellectual growth (Hotez *et al*., 2004). New control strategies will require an understanding of the molecular biology of hookworm infection and the determinants of host specificity. Recognition of a permissive host (PH) is fundamental to completing the life cycle, survival and transmission. For the parasite to successfully colonize a host, infective third-stage hookworm larvae (iL3) must arrive in a PH that will provide nutrients for growth and reproduction. A PH is also susceptible to the parasite by the inability to generate an effective immune response to stop development and expel the worm. A non-permissive host (NPH), however, prevents development and may expel the L3s or drive them into hypobiosis, where they remain infective for a PH when it becomes accessible (Schad, 1990).

The difference between a PH and an NPH might be a protective mechanism in the NPH in response to physical invasion or response to parasite excretory/secretory (ES) products. Host recognition might also be mediated by a positive signal consisting of a specific receptor–molecule interaction between the parasite and its host that determines specificity. Consequently, examining the molecular cross talk between the host and L3s would provide insight into host specificity mechanisms.

As human helminths have complex life cycles and infect niche organs such as lungs and intestine that are not readily accessible, it is challenging to study hookworm infections under laboratory conditions. Parasites can range from strict specialists able to infect only closely related host taxa, to generalists that can infect several unrelated species. The genus *Ancylostoma* offers a gradient across this spectrum, with *A. ceylanicum* as the generalist that can infect dogs, cats, humans and hamsters (Chowdhury and Schad, 1972; Ray and Bhopale, 1972). *Ancylostoma caninum* is a specialist with canids being the primary hosts (Bowman *et al*., 2010). A less strict specialist is *A. duodenale* which infects humans, but have also shown to successfully complete its life cycle in immunosuppressed dogs (Schad, 1979). Species of *Ancylostoma* can infect their host both orally and percutaneously. If hookworm L3s infect by penetrating the skin, they will not resume development until they reach the small intestine (Hawdon *et al*., 1993). If the hookworms are ingested, however, they will develop within the first 24 h in the small intestine.

The aim of the present study was to utilize PH and NPH to identify the differentially expressed genes (DEGs) in response to *A. ceylanicum.* It was hypothesized that an early immune response exists in an NPH in response to invasion and/or ES products, providing a protective mechanism against parasites that does not exist in a PH. It was also hypothesized that the PH provides a positive signal to the parasite in response to invasion or larval stage ES products that initiates development. Herein, tissue-specific gene expression in the small intestine of PH and NPH across 4 different time points early in infection with *A. ceylanicum* was investigated. Enrichment analysis was performed on DEGs across these time points to identify functional responses to hookworm infection. Several DEGs that are candidates for an immune response in mice, as well as permissive signals in hamsters, were discovered. These results contribute to an enhanced understanding of the interaction of *A. ceylanicum* with its host and the role of potential genes critical for host immunity and specificity.

## Materials and methods

### Isolation of hookworm L3

An Indian strain of *A. ceylanicum* (USNPC No. 102954) was maintained in *Mesocricetus auratus*, and hamster feces were collected as described in Bernot *et al*., (2020). Briefly, hamsters with patent infections were moved to cages with wire floors over moistened cardboard to collect feces, which were combined with water and bone charcoal (Ebonex, USA) to create a wet mixture. This mixture was transferred to Petri dishes containing dampened filter paper, and incubated at 28°C for a minimum of 7 days. After 7–10 days, infective larvae were recovered from the coprocultures by a modified Baermann technique. Recovered L3s were transferred to a 15 mL centrifuge tube, then washed twice by centrifugation in nematode handling buffer (BU) (50 mm Na_2_-HPO_4_, 22 mm KH_2_PO_4_, 70 mm NaCl, pH 6.8) (Hawdon and Schad, 1991). Finally, the L3s were resuspended in 10 mL of BU buffer and transferred to a 25 mL tissue culture flask. This flask was stored in a drawer for up to 4 weeks at room temperature until L3s were ready to be used for infection. Prior to infection, living L3s were separated from dead L3 and other debris as described (Huynh *et al*., 2022).

### Animal infection and collection of small intestines

Four groups of 3 hamsters (AURA strain) and outbred mice (Swiss Webster) were infected orally with 250 *A. ceylanicum* L3s in 100 *μ*L BU buffer using a gavage needle. Infected animals were euthanized at 16, 24 and 36 h post-infection. Uninfected animals were used as the 0 h control. Immediately following euthanasia, the small intestines were removed, transferred to a 50 mL tube on dry ice and liquid nitrogen was added. Once the liquid nitrogen evaporated, the snap-frozen intestines were transferred to a −80°C freezer for storage until RNA extraction.

### RNA extraction and preparation for sequencing

Mortars and pestles were cleaned with bleach, washed with RNase-free water and autoclaved. Surfaces were treated with RNaseZAP (Sigma-Aldrich, St. Louis, Missouri, U.S.A.) before processing each sample. Extraction was performed using the RNEasy Maxi Kit (Qiagen), with reagents prepared according to the manufacturer's instructions. The sterile mortar and pestle were placed in dry ice, and 25–50 mL of liquid nitrogen were added to pre-chill the mortar and pestle. An approximately 5–6 cm piece of the proximal end of the small intestine was added to the cold mortar, additional liquid nitrogen was added and the tissue was gently ground to a fine powder. Additional liquid nitrogen was carefully added if needed. One gram of powdered intestine was transferred to a 50 mL centrifuge tube and 15 mL of lysis buffer RLT with *β*-mercaptoethanol was added immediately. This mixture was homogenized using a 21-gauge needle and 10 mL syringe. All the following steps were performed according to the manufacturer's protocol, with all centrifugations done at 3310 × ***g*** and maximum volumes for elution (final elution volume 1.2 mL). For all samples, except the first replicate of hamster, 0, 16, 24 and 36 h time points, DNase was added according to Appendix E of the kit protocol. Eluted RNA was frozen in a −80°C freezer in several aliquots.

For each sample, 1 *μ*L aliquot was thawed to determine the concentration of RNA. A dilution was prepared of 1 *μ*L of sample and 9 *μ*L of nuclease-free water. One microlitre of the diluted and 1 *μ*L of the undiluted samples were measured using a Nanodrop ND-1000 (ThermoFisher Scientific, USA) to determine the RNA concentration. The remaining sample from each aliquot was used for gel electrophoresis on a 1% bleach gel (Aranda *et al*., 2012) to check the integrity of the samples. For each sample, the appropriate volume containing 5 *μ*g of RNA was transferred to a new 1.5 mL tube and nuclease-free water was added to reach a volume of 50 *μ*L. Samples were frozen on dry ice, the tubes sealed with Parafilm and shipped to the Institute of Genome Sciences at University of Maryland Baltimore for sequencing.

### Library construction and sequencing

Strand-specific, dual unique indexed libraries for sequencing on all Illumina platforms were made using the NEBNext^®^ Ultra™ II RNA Library Prep Kit for Illumina^®^ (New England Biolabs, Ipswich, MA, USA). The manufacturer's protocol was modified by diluting adapter 1:30 and using 3 *μ*L of this dilution. The size selection of the library was performed with AMPure SPRI-select beads (Beckman Coulter Genomics, Danvers, MA, USA). Glycosylase digestion of adapter and second strand was done in the same reaction as the final amplification to avoid further cleanups. Sample input for this method was PolyA enriched RNA. The resulting libraries were sequenced on an Illumina NovaSeq 6000 paired-end 100 bp run. Raw sequence reads can be found at https://www.ncbi.nlm.nih.gov/bioproject/934879.

### Bioinformatic analyses

Raw sequencing reads generated for each sample were analysed using the CAVERN transcriptomics analysis pipeline (Shetty *et al*., 2019). Read quality was assessed using the FastQC toolkit (Andrews, 2010) to ensure quality reads for downstream analyses. Reads were aligned to the mouse reference genome GRCm38 or the hamster reference genome MesAur1.0 (available from Ensembl repository) using HISAT2, a fast splice-aware aligner for mapping next-generation sequencing reads (Kim *et al*., 2015). Reads were aligned using default parameters to generate the alignment BAM files. Read alignments were assessed to compute gene expression counts for each gene using the HTSeq count tool (Anders *et al*., 2015) and the mouse reference annotation (GRCm38) or the hamster reference annotation (MesAur1.0). The raw read counts were normalized for library size and dispersion of gene expression. The normalized counts were utilized to assess differential gene expression across time using the R package ‘DESeq2’ (Love *et al*., 2014). *P* values were generated using a likelihood ratio test implemented in DESeq2 and then corrected for multiple hypothesis testing using the Benjamini–Hochberg correction method. Significant DEGs between conditions were determined using a false discovery rate of 5% and a minimum log2 (fold change) of ±1. The filtered set of genes were further utilized to assess the enrichment of gene ontology (GO) terms.

### Gene ontology enrichment analysis

**Fig. 1.**
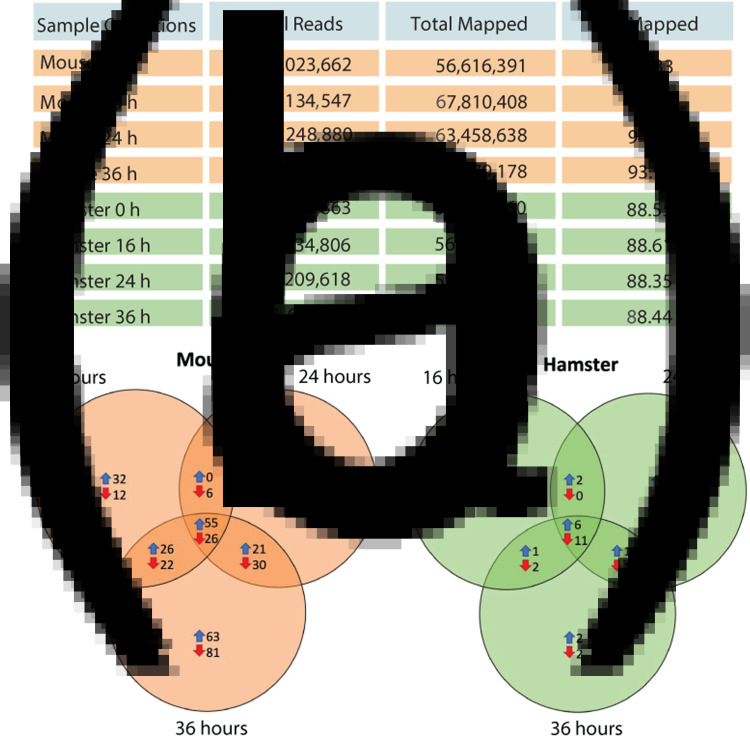
Transcriptome analysis of host responses to *A. ceylanicum* infection. (a) Transcriptome summary with number and percentage of reads mapped to the mouse and hamster genomes when infected with *A. ceylanicum* across time. (b, c) Venn diagram showing the upregulated and downregulated DEGs at 16, 24 and 36 h in (b) mouse and (c) hamster. Upregulated and downregulated DEGs represent a minimum log2 (fold change) of ±1 for each time point, and an adjusted *P* value < 0.05 across all time points.

**Fig. 2.**
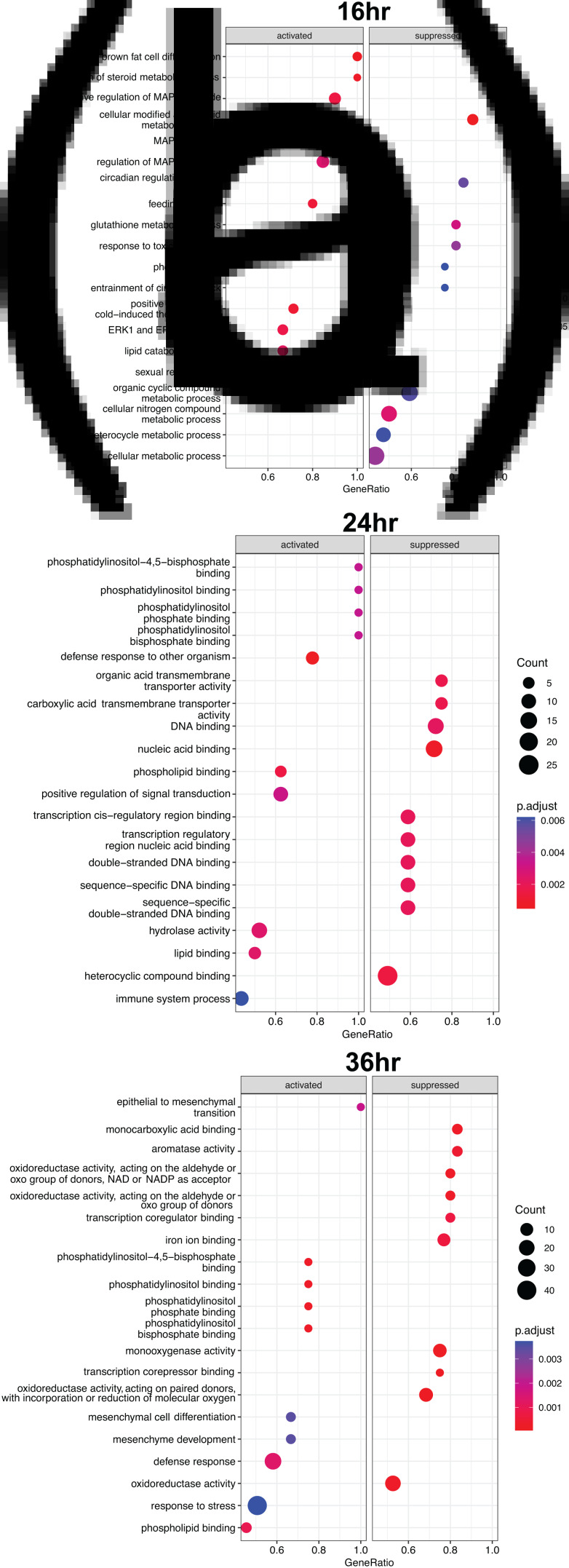
Gene set enrichment analysis of a non-permissive host during infection. (a) GO enrichment analysis of suppressed (downregulated) and activated (upregulated) DEGs at 16 h, (b) 24 h and (c) 36 h post-infection with *A. ceylanicum*. The colour represents the *P*-adjusted value, and size represents the number of genes that fall into each GO category. GeneRatio is the fraction of genes with the given GO term.

**Fig. 3.**
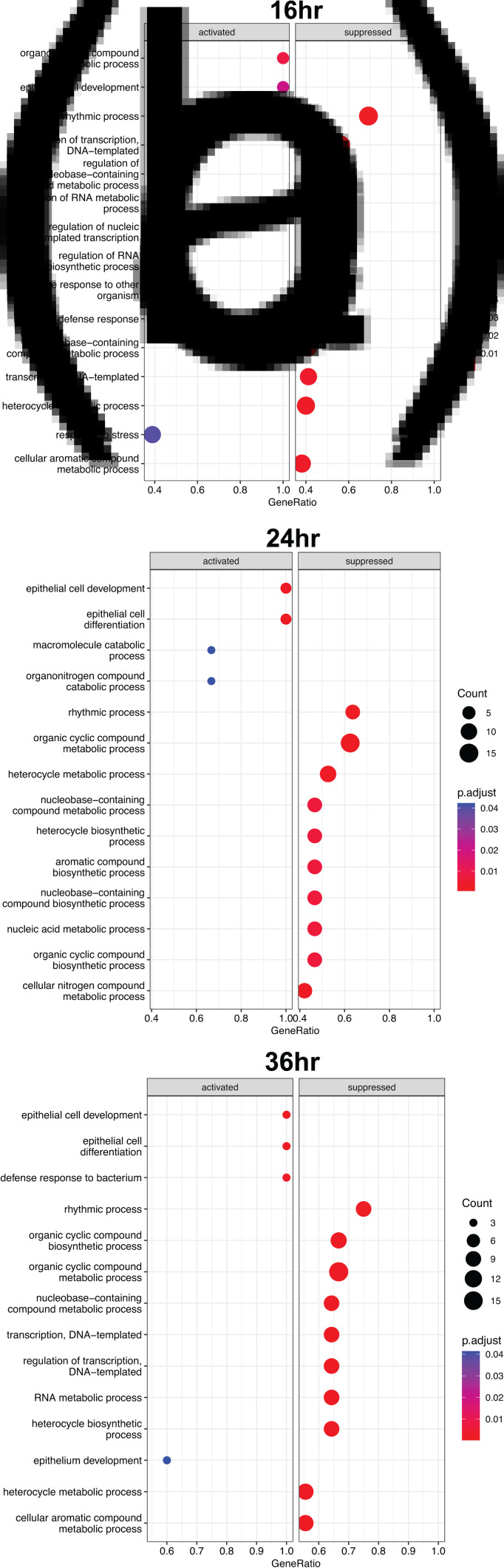
Gene set enrichment analysis of a permissive host during infection. (a) GO enrichment analysis of suppressed (downregulated) and activated (upregulated) DEGs at 16 h, (b) 24 h and (c) 36 h post-infection with *A. ceylanicum*. The colour represents the *P*-adjusted value, and size represents the number of genes that fall into each GO category. GeneRatio is the fraction of genes with the given GO term.

**Fig. 4.**
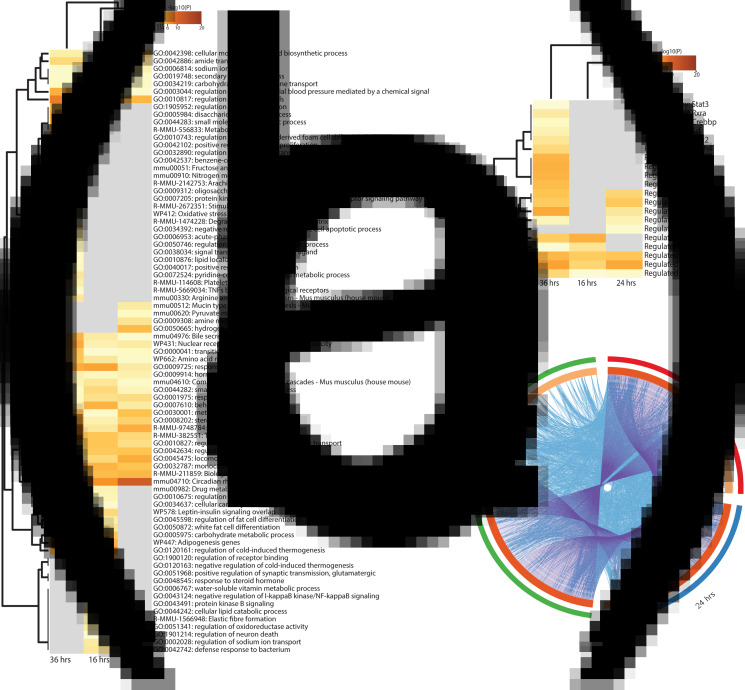
Enrichment analysis and overlap of statistically enriched GO terms in a non-permissive host. (a) Heatmap of enrichment analysis of statistically enriched GO terms for each infection time point. (b) Heatmap of enrichment analysis of all statistically enriched transcription factor targets for each infection time point. (c) Circos plot showing identical DEGs (purple) and GO term overlap (blue) across time points. Outer arcs represent the listed time points, while dark orange arcs represent DEGs that hit multiple time points. Gene ontologies that are unique to a time point are shown under the light orange arc.

**Fig. 5.**
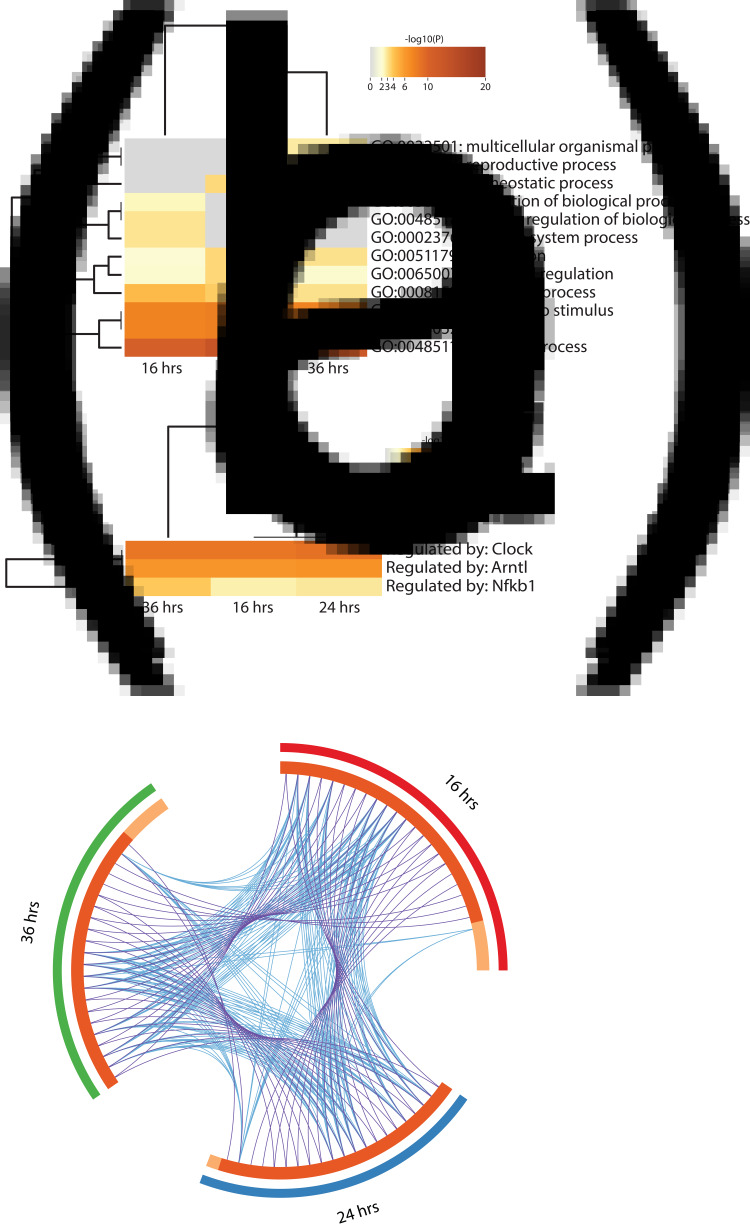
Enrichment analysis and overlap of statistically enriched GO terms in a permissive host. (a) Heatmap of enrichment analysis of statistically enriched GO terms for each infection time point. (b) Heatmap of enrichment analysis of all statistically enriched transcription factor targets across various time points. (c) Circos plot showing identical DEGs (purple) and GO term overlap (blue) across time points. Outer arcs represent the listed time points, while dark orange arcs represent DEGs that hit multiple time points. Gene ontologies that are unique to a time point are shown under the light orange arc.

DEGs across the 4 different time points were visualized using Venny 2.1.0 (Oliveros, 2007). To characterize GO terms across time points, plots were generated in R version 4.2.0 using ClusterProfiler (4.2.2). Moreover, ClusterProfiler enrichment analysis was run with the computational method Gene Set Enrichment Analysis (GSEA) with a *P* value cut-off at 0.05. R packages utilized to visualize the enrichment analyses were ggplot2 (3.3.6), pathview (1.34.0), enrichplot (1.14.2) and ggridges (0.5.3). Circos plots and heatmaps were generated using Metascape (Zhou *et al*., 2019). DEG visualization (Fig. 1) and enrichment heat maps and circos plots (Figs 4 and 5) were generated using a minimum log2 (fold change) of ±1 for each individual time point, and adjusted *P* values < 0.05. Enrichment analyses (Figs 2 and 3) were generated using a minimum log2 (fold change) of ±1 for each individual time point to explore additional gene ontologies.

## Results

### *Ancylostoma ceylanicum* produce distinct transcriptional profiles in permissive and non-permissive hosts

Transcriptomic profiles were generated for the small intestine in mice and hamsters at 4 different time points, each with 3 biological replicates; 0, 16, 24 and 36 h post-infection, resulting in ~2000 DEGs. Mouse replicates had substantial differences between each time point while variation was observed in the hamster replicates within time points (Fig. S1). Reads mapped to the genomes were >88% for hamsters and >93% for mice across all time points (Fig. 1a). Mice exhibited a greater number of DEGs overall compared with hamsters. At 36 h, the highest number of DEGs for mice was identified, which were 63 upregulated DEGs and 81 downregulated DEGs (Fig. 1b). In hamsters, however, the number of DEGs was similar and low across all separate time points, ranging from 0 to 3 for both up- and downregulated DEGs. In hamsters, a core set of DEGs in the nexus of all time points was observed: 6 upregulated and 11 downregulated DEGs (Fig. 1c).

### GO enrichment analysis and global transcriptome changes during infection

To identify the major functional categories represented by the DEGs, GO enrichment analysis was performed on each time point for both species. At 16 h post-infection, the top activated biological processes in mice were brown fat cell differentiation and regulation of steroid metabolic process (GO:0050873 and GO:0019218) (Fig. 2a). In hamsters at 16 h post-infection, several defence responses were activated (e.g. GO:0006952/defence response, GO:0006950/response to stress and GO:0098542/defence response to other organisms) (Fig. 3a). For both species, these GO terms were no longer enriched at the 24 and 36 h time points (Figs 2b, c and 3b, c).

At 24 h post-infection, mice displayed 2 activated gene ontologies important in response to other organisms and immune stimuli (e.g. GO:0098542/defence response to other organism, and GO:0002376/immune system process) (Fig. 2b). There were no immune responses/responses to stimuli activated in hamsters at 24 h. Instead, the top activated GO terms were GO:0002064/epithelial cell development and GO:0009057/macromolecule catabolic process (Fig. 3b).

At 36 h post-infection, mice exhibited similar activated GO terms to what was discovered at 16 h post-infection in hamsters (GO:0006950/response to stress, GO:0006952/defence response) (Fig. 2c). In hamsters, the activated genes at the 36 h time point were epithelial cell development (GO:0002064) and defence response to bacteria (GO:0042742) (Fig. 3c).

Next, the top enrichment clusters for both species were examined, and it was found that mice have a higher number of enriched GO terms (Figs 4a and 5a). For early infection stages, at 16 h only, mice were found to have enriched GO terms that are potentially important in a defence response to *A. ceylanicum* (e.g. GO:0048545/response to steroid hormone, GO:0042742/defence response to bacterium and GO:1900120/regulation of receptor binding) (Fig. 4a). Surprisingly, hamsters did exhibit enriched GO:0002376/immune system process at 16 h, even though they are a PH. In addition, hamsters did have enriched GO:0050896/response to stimulus consistently across all 3 time points (Fig. 5a). Mice had several crucial GO terms in response to infection, but these showed up in later infection stages; at 36 h (e.g. R-MMU-2672351/stimuli-sensing channels, GO:0042102/positive regulation of T-cell proliferation, R-MMU-5669034/TNFs bind their physiological receptors and GO:0007205/protein kinase C-activating G protein-coupled receptor signalling pathway). Finally, GO:0009725/response to hormone was significantly enriched at all time points in mice (Fig. 4a).

The enrichment of targets from conserved transcriptional regulators was also examined. Enriched targets from 20 transcription factors in mice were found, in which 13 of these are potentially related to an immune response (*Nfe2l2*, *Nr1i3*, *Nr1i2*, *Nr1d1*, *Nr1h4*, *Rxra*, *Cdx2*, *Srf*, *Crebbp*, *Stat1*, *Stat3*, *Sp1* and *Rela*) (Fig. 4b). In hamsters, enriched targets from 3 transcription factor hits were present, all of which may play a role in immune function (Fig. 5b). Both species had increasing expression values for *nfkb1* gene targets across time, though hamsters had a slightly more enriched response at all time points [e.g. 4 *vs* 3-log10(*P*) at 36 h] (Figs 4b and 5b). Targets from the circadian rhythm target genes, *Clock* and *Arntl*, which control the temporal gating of cytokine production and chemokine attraction (Vieira *et al*., 2020), did not change across the time points in hamsters but had a slight increase across time in mice.

Overlapping DEGs and GO terms across time in mice and hamsters were visualized with Circos plots, showing that mice have a higher overlap of DEGs and GO terms across all time points. Mice also showed a higher amount of DEGs and new GO functions at 36 h, compared to hamsters (Figs 4c and 5c).

### Gene-expression patterns of candidate genes responsible for protection and host specificity

**Fig. 6.**
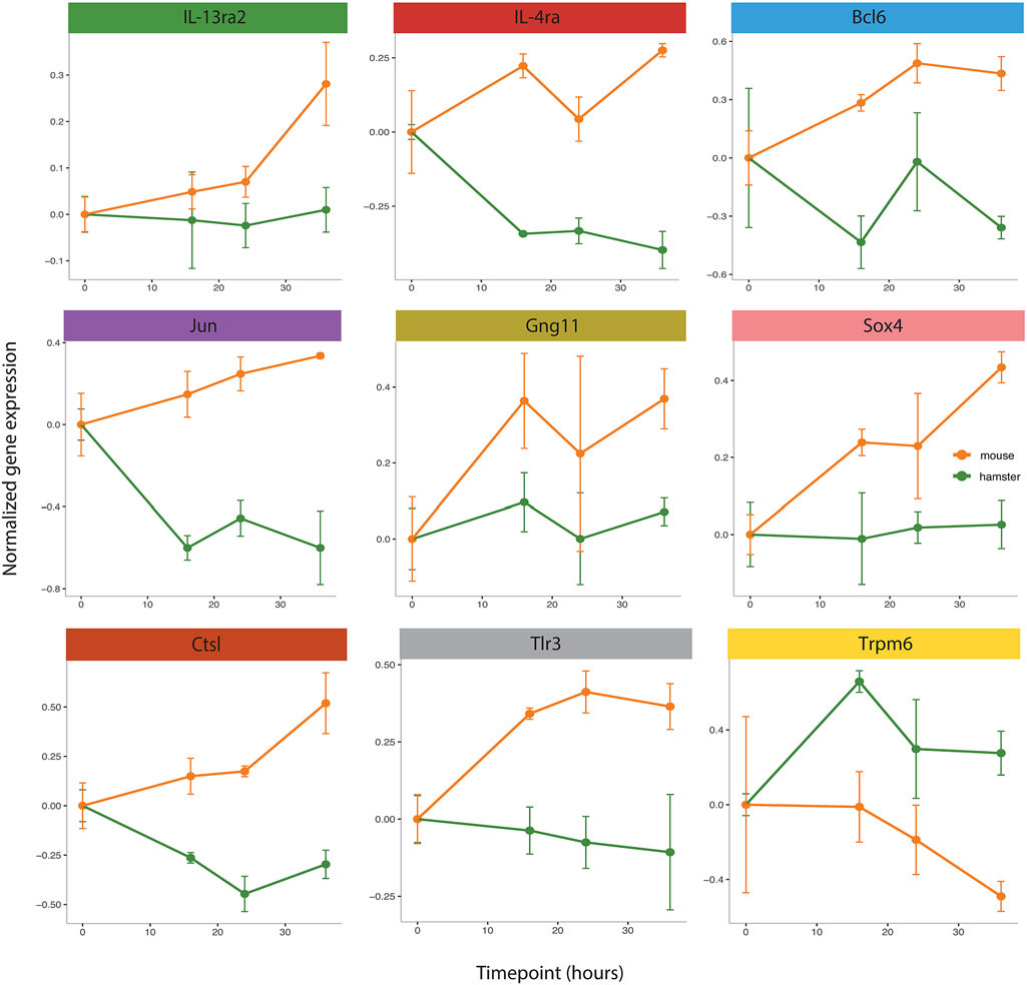
Transcriptome analysis of candidate DEGs responsible for infection outcome. Averaged normalized gene expressions from 4 time points with 3 mouse (orange) and 3 hamster (green) samples per time point (0, 16, 24 and 36 h). Error bars represent the standard deviation. Each gene colour correlates to their location in the proposed mechanism against hookworms shown in Fig. 7.

Hypothesis-driven analyses were carried out based on current knowledge about the host immune response and the potential infection strategies of hookworm that may play a role in determining host specificity. By considering the host immune system evasion strategies of *A. ceylanicum* and other soil-transmitted nematodes (STNs), gene candidates that should be investigated further were identified. Here, DEGs included have adjusted *P* values < 0.05, while all log fold changes (LFCs) were included to investigate additional biological processes that may play an important role in protection and host specificity. Moreover, this included analysing the differences between the PH and NPH's immune systems such as T- and B-cell responses and their effector molecules, cytokines, tuft cell profiles and other G protein-coupled receptors. Hence, the exploration of whether the Th2 response responsible for parasite expulsion differs in the PH and NPH. The Th2 genes causing the ‘weep and sweep’ model of smooth-muscle cell contractility, intestinal permeability and RELM-beta secretion were more active in mice than hamsters. For instance, in non-permissive mice, the LFC of *interleukin-13 receptor subunit alpha-2* (*Il13ra2*) was 3.32 at 36 h compared to the 0 h control (Fig. 6 and Table S2). *Il13ra2* expression in permissive hamsters, on the other hand, had no significant change. In addition, *interleukin-4 receptor subunit alpha* (*Il4ra*) was upregulated in mice and downregulated in hamsters, with LFC values of 0.40 and −0.65 at 36 h, respectively (Fig. 6 and Table S2).

Gene candidates for a T follicular helper response were also investigated. Interestingly, the transcription factor *B-cell lymphoma 6 protein homolog* (*Bcl6*) was significantly upregulated in the non-permissive mice with an LFC value of 0.71 at 36 h (*P* < 0.001). In permissive hamsters, *Bcl6* was downregulated with an LFC value of −0.93 (Fig. 6 and Table S2). The *transcription factor jun* (*Jun*) found to regulate Th1 and Th2 responses was upregulated in mice with an LFC value of 0.48, and downregulated in hamsters with an LFC value of −0.98 at 36 h (Fig. 6 and Table S2).

Next, G proteins and tuft cells were investigated due to their known role in response to helminths. *Guanine nucleotide-binding protein gamma 11* (*Gng11*) had an increase in gene expression at 36 h in mice (LFC = 0.66) and remained unchanged in hamsters (Fig. 6 and Table S2). The *transcription factor sox-4* (*Sox4*) expression had an LFC of 0.66 at 36 h in mice, while no change was detected in hamsters (Fig. 6 and Table S2).

Other protective genes were also examined and dissimilar patterns between the 2 species were found for *cathepsin L1* (*Ctsl*) and *toll-like receptor 3* (*Tlr3*). Both genes were upregulated in non-permissive mice with LFCs of 0.77 and 0.55, respectively (Fig. 6 and Table S2). *Tlr3* was not significantly changed in the permissive hamsters, while *Ctsl* was downregulated with an LFC of −0.51 (Fig. 6 and Table S2).

Finally, pathways upregulated in the PH were examined to search for a potential positive signalling pathway that informs the parasite that it is in the correct host environment. *Transient receptor potential cation channel subfamily M member 6* (*Trpm6*), *transmembrane and immunoglobulin domain-containing protein 1* (*Tmigd1*) and *lysophospholipid acyltransferase 1* (*Mboat1*) were all downregulated in the non-permissive mouse host and upregulated in the permissive hamster host (Tables S1 and S2). Moreover, *Trpm6* gene expression was increased early in hamsters (LFC = 1.07 at 16 h) and decreased in mice across time, eventually with an LFC of −1.12 at 36 h (Fig. 6 and Table S2). Additional upregulated genes in permissive hamsters were *immunoglobulin heavy variable 1–53* (*Ighv1–53*), which had a surprisingly high LFC of > 4.5 at 16 and 36 h in hamsters (Table S2). Finally, *nitric oxide synthase* (*Nos2*) was downregulated in mice but there was no change found in hamsters (Tables S1 and S2).

## Discussion

The immune response to hookworm is complex and variable, but studies on *Necator americanus* hookworm infections in naive healthy human volunteers have revealed eosinophilia, parasite-specific IgG and IgE and secretion of both Th1 [interferon (IFN)-*γ* and tumour necrosis factor (TNF)-*α*] and Th2 [interleukin (IL)-5 and IL-13] cytokines (Maxwell *et al*., 1987; Wright and Bickle, 2005; Geiger *et al*., 2008). Interestingly, an increase of IL-10 was discovered during larval migration, at arrival in the intestinal tract, and at the end of the pre-patency period, which may play an important role in order to minimize intestinal inflammation and effective type 2 response in favour of the worm (Geiger *et al*., 2008). Another study of infected individuals in Papua New Guinea, where *N. americanus* is highly endemic, confirms that hookworms induce a mixed Th1/Th2 response (Quinnell *et al*., 2004). Moreover, this study suggests that IFN-*γ* mediates protection against adult worms, but is suppressed by established adult worms, suggesting a negative effect of the IFN-*γ* response on the worm (Quinnell *et al*., 2004). The mucosal and systemic immune response to hookworm in healthy humans has also shown to result in a strong Th2 response, and some evidence of a Th1 response (Gaze *et al*., 2012). Here, it was evident that Th2 cytokines are present in the non-permissive mice through the upregulation of their receptors; *Il13ra2* and *Il4ra*. Interestingly, the IL-4R*α*/signal transducer and activator of transcription 6 (STAT-6) is activated by both IL-4 and IL-13 (Horsnell *et al*., 2007). Moreover, IL-4R*α*-deficient mice have delayed expulsion of *Nippostrongylus brasiliensis* due to decreased Th2 cytokine production in the mesenteric lymph node, and delayed intestinal goblet cell hyperplasia. One protein secreted by *N. americanus*, NKBP, binds to natural killer cells and induces the production of IFN-*γ*. This suggests that the worm skews a Th2 deleterious response into a Th1 response, contributing to worm long-term survival (Hsieh *et al*., 2004), which might be happening in permissive hamsters in this study.

Anti-helminth immunity depends on B cells and the help of T follicular helper cells in regulating the type 2 immune response by isotype switching to parasite-specific IgG (Weinstein *et al*., 2012). The transcription factor BCL6 is necessary for T follicular helper cell differentiation, and T cell help to B cells in mice (Johnston *et al*., 2009; Nurieva *et al*., 2009; Yu *et al*., 2009). *Ancylostoma* secreted protein-2 from *N. americanus* (*Na*-ASP-2) suppressed B-cell receptor signalling using a human proteome microarray, particularly by downregulating the transcription of *lyn* and *pi3k*, which interact with CD79A on B cells (Tribolet *et al*., 2015). *Na*-ASP-2 is secreted by larvae upon entry into their host. This protein has structural similarities to some chemokines, suggesting that ASP-2 is a ligand for an unknown receptor, and implies that it modulates the host immune response (Asojo *et al*., 2005). ASPs are also secreted by the canine hookworm *A. caninum* L3s during early infection, including *Ac*-ASP-1 (a single domain protein) and *Ac*-ASP-2 (a double domain protein) (Hawdon *et al*., 1996, 1999). The upregulation of *Bcl6* in non-permissive mice suggests that the ASPs may only be able to successfully inhibit B-cell signalling in PHs, while the NPH can upregulate its B-cell response following infection.

The AP-1 transcription factor JUNB is known to regulate both type 1 and type 2 immune responses in response to Toll-like receptors, which will positively regulate macrophages, and in return will either promote the classical M1 polarization, or IL-4, which will activate the alternative M2 phenotype (Fontana *et al*., 2015). Moreover, JUNB deficiency in *N. brasiliensis* compromises type 2 activation during infection, leading to lowered cytokine production and eosinophil recruitment and increased parasite burden (Fontana *et al*., 2015). The importance of *Jun* upregulation in non-permissive mice in this study is also supported by the upregulation of the *Cdx2* target genes, which are activated in the MAPK pathway by *jun* and other genes (Coskun *et al*., 2011). CDX2 is known to play a role in maintaining intestinal homoeostasis and is activated by inflammatory cytokines, either through the MAPK signalling or the nuclear factor kappa B (NF-*κ*B) signalling pathway (Coskun *et al*., 2011). This also suggests that only non-permissive mice can respond appropriately to infection by upregulating immune response genes.

Next, intestinal epithelial tuft cells were explored as they have been demonstrated to play an essential role in initiating type 2 immunity (Coakley and Harris, 2020). A mechanism has been proposed where tuft cells detect the presence of helminths through G protein-coupled receptors, which elicit the release of IL-25 and leukotrienes, driving type 2 innate lymphoid cells (ILC2) activation. In a positive feed forward loop, ILC2-derived IL-13 will promote tuft and goblet cell differentiation and hyperplasia, ultimately expelling the worm (Howitt *et al*., 2016; von Moltke *et al*., 2016). However, this immune response clearly fails to provide protection and eradication of most helminth infections. This suggests that the parasites achieve a homoeostatic state where excess morbidity is avoided, and complete elimination of the worms prevented. Hence, G protein-coupled receptors and their G-proteins were investigated due to their possible role in parasite protection as a dominant mechanism in tuft cells regulating immune responses (Schneider *et al*., 2019). *Guanine nucleotide-binding protein G*(*T*) *subunit gamma-T1* (*Gngt1*) had the highest LFC of all genes analysed with a value of ~5 across all 3 time points in the NPH, while unchanged in the permissive hamster host (Table S2). Other G proteins were *guanine nucleotide-binding protein gamma 2* (*Gng2*) and *Gng11*, which were both upregulated in mice while there was no change in hamsters.

Tuft cells were expected to be upregulated in an NPH, but the known tuft cell markers *arachidonate 5-lipoxygenase-activating protein* (*Alox5ap*), *serine/threonine-protein kinase* (*Dclk1*) and *transient receptor potential cation channel subfamily M member 5* (*Trpm5*) were downregulated in the NPH (Tables S1 and S2). Still, *Alox5pa* was eventually upregulated at the 36 h time point in mice. *Trpm5* was only downregulated at 16 h, then showed negligible change at 24 and 36 h in mice. Interestingly, the tuft cell *neuronal regeneration-related protein* (*Nrep*) and *Sox4* were increasingly upregulated over time in non-permissive mice, potentially contributing to protection against infection. The function of SOX4 is unknown, but it has been shown to promote secretory differentiation in the absence of ATOH1 (Gracz *et al*., 2018). *Sox4* knockout mice had impaired tuft cell hyperplasia and parasite clearance when infected with *N. brasiliensis*. Even though not all tuft cell genes had the expected patterns across the 2 species, the early downregulated tuft cell genes in the NPH were still becoming less downregulated, and slightly increased, across time. This coincides with the expected time of a tuft cell response.

Excretory–secretory products (ESPs) are important for hookworm survival and modulation of host immune responses, suggesting that host recognition of these is crucial for host immunity. The upregulation of *Ctsl* and *Tlr3* in the NPH might initiate the necessary immune response. TLR3 is involved in the recognition of nucleic acid-like structures, and proteases released from helminths block the MyD88-independent TRIF-dependent signalling pathway by degradation of TLR3s (Akira and Takeda, 2004; Donnelly *et al*., 2010). This suggests that non-permissive mice possibly compensate for the loss of TLR3s by upregulating its gene expression. The differences in *Ctsl* transcription in these data may show the important role of cysteine proteases in combating parasitic infections. For instance, *A. caninum* is found to express a haemolytic protein in the gut during both adult and larval stages. As *A. caninum* digest whole erythrocytes, the haemolysin in the worm's intestine forms pores in the erythrocyte membranes so that haemoglobin can be released (Don *et al*., 2004). *Cathepsin L* also plays a role in antigen processing, suggesting that only the non-permissive mice are able to process exogenous proteins effectively during infection prior to the activation of CD4 + T cells (Zhang *et al*., 2001).

The ability to process ESPs is also evident due to the enrichment of *Nr1i3* and *Nr1i2*, whose human orthologues function as key regulators in xenobiotic and endobiotic metabolism (Fig. 4b) (Chen *et al*., 2019). Double null mice lines lacking *Nr1i3* and *Nr1i2* have shown that they are not sensitive to a broad range of xenobiotic inducers that normally induce both receptors (Zhang *et al*., 2004). In addition, both receptors induce the expression of several detoxifying enzymes in response to activation, including cytochrome P450, UDP-glucuronosyltransferase, dehydroepiandrosterone sulphotransferase and transporters. Finally, the enrichment of *Nfe2l2* seen only in mice codes for anti-oxidant, anti-inflammatory and detoxifying proteins, suggesting mice are able to process ESPs (Loboda *et al*., 2016).

The *Rxra* gene target, which functions in attenuating the immune system by suppressing type I IFN expression (Ma *et al*., 2014), is only differentially expressed at the 36 h time point in mice. Similar expression for the *Srf* gene target was found, which is an essential transcription factor in haematopoiesis and mature myeloid cell function, and regulates neutrophil migration, integrin activation and trafficking (Taylor and Halene, 2015).

Due to the ability of *A. ceylanicum* to block voltage-gated potassium channels (Chhabra *et al*., 2014), the expression of these genes was also analysed to see whether the PH has fewer of these. From all the potassium channels identified, 6 were upregulated (*Kcnj15*, *Kcnj13*, *Kcnmb1*, *Kcne3*, *Kcnq*1, *Kcnj16*) and 3 were downregulated in mice (*Kcnn4*, *Kcnk5*, *Kcnk10*). The 9 potassium channels did not change significantly in hamsters (Tables S1 and S2). These voltage-gated channels may interact with parasitic ESP. For instance, AcK1 is a peptide expressed in the anterior secretory glands of both *A. caninum* and *A. ceylanicum*. The peptide binds to native Kv1.3 in human T cells, blocking their function and proliferation (Chhabra *et al*., 2014).

As hookworm induces a mixed Th1/Th2 response, differences in Th1 effector molecules between the 2 species were investigated to see the effect of infection on the inflammatory response. Out of the 9 TNF genes identified, 6 of these were upregulated in mice (e.g. *Tnfrsf21*, *Tnfsf10*, *Tnfrsf23*, *Tnfrsf11b*, *Tnfsf13b* and *Tnfaip8l3*) and 3 were downregulated in mice (e.g. *Tnfrsf13b*, *Tnfaip3* and *Tnfsf9*), while all 9 remained unchanged in hamster (Tables S1 and S2). Additional Th1-related effector molecules upregulated in non-permissive mice were *interleukin-5 receptor subunit alpha* (*Il15ra*) and *signal transducer and activator of transcription 2* (*Stat2*). NF-*κ*B genes (e.g. *Nfkbia* and *Nfkbiz*) were downregulated in mice, as well as *interleukin 18* (*Il18*), *interleukin 15* (*Il15*) and *interleukin 34* (*Il34*). None of these Th1 genes changed appreciably in hamsters (Tables S1 and S2).

Chemokines and cytokines were further studied to compare the Th1 and Th2 effector responses in the 2 species as these determine the nature of the immune response. The Th1/Th2 chemokines 6 and 8 were the only cytokine/chemokine genes upregulated in the PH, while there was no applicable change in NPH (Tables S1 and S2). The inflammatory *C-C motif chemokine 2* (*Ccl2*), *C-C motif chemokine 25* (*Ccl25*) and *C-X-C chemokine receptor type 4* (*Cxcr4*) were downregulated in the NPH, and unchanged in the PH. Yet, several chemokine genes were upregulated in non-permissive mice while there was no significant change in permissive hamsters (e.g. *Ccr1*, *Ccr2*, *Ccr5* and *Ccl22*).

The GO enrichment analysis also indicates that Th1 and Th2 processes are enriched in an NPH (Fig. 5b). This is apparent by the enriched STAT1 and STAT3 targets in mice only, which are known to be necessary for optimal Th1 differentiation and B-cell proliferation, respectively (Levy and Lee, 2002; Ma *et al*., 2010). The *Crebbp* activation, seen at 36 h after infection in mice, may also play a role in the mixed T-cell effector response due to CREB's role in proliferation, survival and differential regulation of Th1, Th2 and Th17 responses (Wen *et al*., 2010). CREB is also required for the generation and maintenance of regulatory T cells, and inhibits NF-*k*B activation. The production of chemokine CXCL4 is induced by the activation of Sp1 intrinsic innate immune responses and the recruitment of inflammatory cells (Dupuis-Maurin *et al*., 2015). RELA (NF-*κ*B p65) gene targets were also being differentially expressed, and its phosphorylation is associated with enhanced transcriptional activity of NF-*κ*B (Chen and Greene, 2004). Finally, NR1H4 regulates inflammatory responses and barrier function in the intestinal tract, while *Nr1d1*'s transcription, though a rhythm gene, has been found to upregulate inflammation cytokines (Il-6 and COX-2) and nuclear translocation of NF-*k*B (Attinkara *et al*., 2012; Xiang *et al*., 2021). Regulation of barrier function in the NPH is also evident by the upregulation of *transcription factor gata-5* (*Gata5*) in mice and the lack of appreciable change in hamsters (Tables S1 and S2). GATA5 is responsible for activation of several intestinal genes, and its inactivation in mice results in vascular endothelial dysfunction and hypertension (Messaoudi *et al*., 2015). In these results, the NPH can regulate proinflammatory cytokines, which confirms the mixed T effector cell response to parasites, and that its regulation is evidently more significant in an NPH.

The anti-inflammatory T regulatory cell (Treg) response was also explored to see if it differed between the 2 species due to the benefits for a parasite during host immune suppression. Treg-mediated immunosuppression in the human host through cytokines such as IL-10 and transforming growth factor (TGF-*β*) dampens inflammation and restores homoeostasis, which contributes to the failure of the immune system to clear the parasites (Velavan and Ojurongbe, 2011). Here it was found that the expression of immune suppression genes was not uniform in mice and hamsters (e.g. *Tgfb1i1*, *Tgfbi* and *Tgfbr3*, and *Il10ra*). Tregs are also dependent on the expression of 5′-nucleotidase (NT5E or CD73) (Kordaß *et al*., 2018), which was upregulated in the permissive hamster and did not change significantly in the non-permissive mouse (Tables S1 and S2). *Necator americanus* is found to exhibit anti-inflammatory proteins within their ES products named AIP-1 and AIP-2 that are members of the tissue inhibitor of metalloproteases (TIMP)-like proteins. Studies on *Ac*-AIP-1 in a TNBS colitis model show suppression of colon IL-10, TGF-*β* and TSLP and Treg cell accumulation, making the protein a novel therapeutic candidate for inflammatory bowel disease treatment (Ferreira *et al*., 2017). Recent studies on *Na*-AIP-1 show that prophylactic *Na*-AIP-1 in mouse models downregulates signalling pathways involved in type-1 inflammation, particularly TNF (Buitrago *et al*., 2021). Moreover, *Na*-AIP-1 co-culture with human monocyte-derived M1 macrophage cell line also resulted in significantly reduced secretion of TNF. Here, a more significant suppression of IL-10RA in the PH was seen, but there were mixed results for different TGF-*β* genes in mice (Tables S1 and S2). NT5E, which is found to be expressed on Treg cells and may play a role in restricting anti-inflammatory responses, was upregulated in the PH (Kordaß *et al*., 2018). Thus, regulation of anti-inflammatory responses seems to be more active in the permissive hamsters, whereas mice might remain unaffected by the *A. ceylanicum* orthologue of Na-AIP-1.

Finally, the possibility of any genes encoding a positive signal in a permissive that may inform the parasite that can resume its developmental processes was considered. This prompted us to evaluate the genes that are upregulated in a PH and downregulated in an NPH. *Trpm6* might act as a positive signalling gene in a PH for the parasite to establish in the host and eventually start feeding. *Trpm6* is targeted in flatworms by the drug praziquantel, triggering Ca^2+^ influx, resulting in spastic paralysis of the worms (Babes *et al*., 2017). The parasite is also dependent on nutrients such as magnesium (Mg) in the host to survive and conduct their biological processes. In fact, Mg deficiency in mice protects them from protozoan parasites (Maurois *et al*., 1995). Interestingly, *Trpm6* in the host regulates Mg balance in the intestine by taking up Mg from the lumen into the blood (de Baaij *et al*., 2012). This suggests that a PH may make more Mg available for parasite feeding and survival in the host, while an NPH prevents this process from occurring. The highest LFC in permissive hamsters was by IGHV1–53, which is predicted to facilitate antigen-binding activity and immunoglobulin-binding activity in mice (Bothwell *et al*., 1981). Though an antibody response is known to target parasites, an early and significant antibody response in a PH suggests that antibody responses have a limited effect. In trematodes, antibody-trapping happens during secretion of parasite ESPs and antibody binding, which happens simultaneously and constantly (Cortés *et al*., 2017). A layer of ESPs that covers the parasite traps surface antibodies which are eventually degraded by parasite-derived proteases. This suggests that antibody trapping might be a host–parasite interaction present in permissive hamsters that plays a role in susceptibility to infection.

Hookworms protect themselves from lethal reactive oxygen species (ROS) with a layer of antioxidants (Callahan *et al*., 1988). ROS are released from both the host immune effector cells and through degradation of haemoglobin. Induced nitric oxide synthase provides protection against *Strongyloides venezuelensis* infection in mice, as inducible nitric oxide synthase (iNOS) knock out (KO) resulted in mice being more susceptible to infection (Rodrigues *et al*., 2018). Previous studies have found that NO can contribute to either parasite clearance or to susceptibility by exacerbating inflammation that worsens disease manifestation, or facilitates parasite killing (Diliani and Dondji, 2017). Here, a downregulation of *Nos2* in the NPH was found, implying that the NPH can avoid heightened inflammation and susceptibility (Tables S1 and S2).

**Fig. 7.**
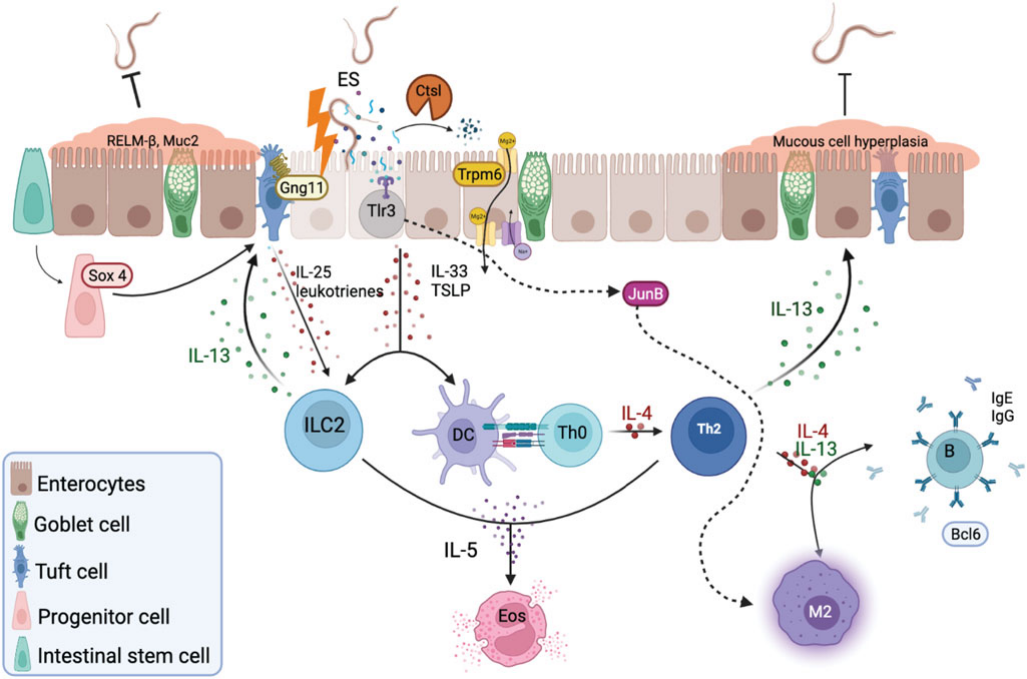
Representative schematic of the small intestine during helminth infection. After larval migration through the lungs and intestinal mucosa, epithelial cells secrete various alarmins in response to intestinal damage and ESPs, including IL-33 and thymic stromal lymphopoietin (TSLP). These products together with IL-25 and leukotrienes produced by G protein-coupled receptor signalling in tuft cells promote the activation and differentiation of type 2 innate lymphoid cells (ILC2) and CD4 T helper 2 (Th2) cells. ILC2-derived IL-13 will promote tuft and goblet cell differentiation and hyperplasia, ultimately expelling the worm. Th2 secretion of IL-4, IL-5 and IL-13 lead to eosinophil activation, differentiation of M2 macrophages, induction of parasite-specific IgG and IgE, and contributes to the smooth-muscle cell contractility, intestinal permeability and RELM-beta secretion. The additional proposed mechanisms here consist of the transcription factor SOX4 which may promote differentiation of tuft cells, while G protein-coupled receptors, such as GNG11, activate tuft cells in response to unknown helminth ESPs. The protease CTSL may downgrade ESPs and process antigens, while TLR3 binds parasitic nucleic acids to activate JUN regulation of Th1 and Th2 response, by either promoting the classical M1 polarization, or IL-4, which will activate the alternative M2 phenotype. Signalling by TRPM6 acts as a positive signal in a permissive host by providing nutrients such as Mg to the parasite to survive and conduct their biological processes within the host.

An understanding of the molecular mechanisms that determine host specificity could play a pivotal role in potential new control strategies of parasitic helminths. Parasite ESPs are believed to modulate their host's immune response, and as more helminth genome sequences are being elucidated, additional ESPs and their role in evading the host immune response are being revealed. These ESPs might also elicit a positive signal from the PH that allows parasites to resume development in the host. *Ancylostoma ceylanicum* was found to produce distinct transcriptomic profiles in PH and NPH which provide insight into potential protective mechanisms. These results suggest that *A. ceylanicum* larvae and their ESPs induce release of alarmins that are only recognized by certain immune gene products in the non-permissive mice, such as G protein-coupled receptors and antigen-presenting cells (Fig. 7). Th2 response genes such as *Il13ra2*, *Il4ra* and *Bcl6* are upregulated in the NPH, suggesting these are crucial genes to fight off infection by induction of parasite-specific IgG and IgE, smooth-muscle cell contractility, intestinal permeability and resistin-like molecule (RELM)-beta secretion (Fig. 7). The ability of mice to upregulate the transcription factor *Sox4* may promote differentiation of tuft cells, while *Gng11* upregulation might drive the activation of ILC2. The antigen-presenting/processing genes *Tlr3* and *Ctsl* are upregulated in mice and may also contribute to the appropriate T-cell effector response together with *Jun* (Fig. 7). The most compelling positive signalling gene in the permissive hamsters were *Trpm6* which might provide the parasite with nutrients in the form of Mg for the larvae to eventually start feeding and other biological processes (Fig 7).

Overall, an increased activity of the immune system in the non-permissive mice was found, while permissive hamsters fail to provide protection, most likely in response to modulating ES products released by *A. ceylanicum*. Here, non-permissive mice regulate genes associated with protection but might also have a higher baseline of protection prior to infection, as suggested by their higher normalized gene expression values. Still, these gene expression data provide novel insight into the molecular determinants of host specificity by recognizing the differences in activated signals and pathways between PH and NPH. Further research is needed to identify the ESPs released by L3s into the different host environments to advance the understanding of the crosstalk between hookworm L3s and their host.

## Supplementary material

For supplementary material accompanying this paper visit https://doi.org/10.1017/S0031182023000227. click here to view supplementary material
