# Supplementary material for: Comparative transcriptomics from intestinal cells of permissive and non-permissive hosts during Ancylostoma ceylanicum infection reveals unique signatures of protection and host specificity
Source: Parasitology. 2023 Mar 8;150(6):511–23. doi: 10.1017/S0031182023000227 (PMC10192101; doi:10.1017/S0031182023000227)
Supplement: Supplementary file 1 [file S0031182023000227sup.zip › S0031182023000227sup002.pdf]

Gene	Average normalized gene expression levels							
	Mouse				Hamster			
	0 hrs	16 hrs	24 hrs	36 hrs	0 hrs	16 hrs	24 hrs	36 hrs
il13ra2	3.23	3.28	3.30	3.51	4.05	4.04	4.03	4.06
il4ra	10.70	10.92	10.74	10.98	10.76	10.42	10.43	10.37
bcl6	8.84	9.13	9.33	9.28	7.43	7.00	7.41	7.07
jun	10.91	11.06	11.16	11.25	10.25	9.64	9.79	9.64
gngt1	2.58	2.71	2.67	2.72	0.00	0.00	0.00	0.00
gng2	8.58	8.78	8.62	8.81	8.06	8.03	8.05	8.09
gng11	7.96	8.32	8.18	8.33	7.17	7.26	7.17	7.24
alox5ap	6.70	6.55	6.59	6.90	6.08	6.05	6.13	6.09
dclk1	8.06	7.73	7.80	7.84	5.92	5.93	5.90	5.91
trpm5	8.07	7.68	8.10	8.04	2.92	2.93	2.93	2.95
nrep	7.00	7.26	6.98	7.32	7.35	7.48	7.34	7.43
sox4	9.81	10.05	10.04	10.25	6.92	6.91	6.94	6.95
ctsl	10.59	10.74	10.76	11.11	9.52	9.26	9.07	9.22
tlr3	10.12	10.46	10.53	10.48	9.88	9.85	9.81	9.78
kcnj15	6.01	6.41	6.33	6.26	7.45	7.30	7.43	7.49
kcnj13	7.48	7.99	8.05	8.56	0.00	0.00	0.00	0.00
kcnmb1	7.10	7.39	7.25	7.45	5.00	4.95	4.97	4.98
kcne3	10.44	10.61	10.88	10.82	9.59	9.77	9.72	9.66
kcnq1	10.33	10.49	10.52	10.66	9.50	9.32	9.36	9.36
kcnj16	4.86	4.83	5.16	5.81	0.00	0.00	0.00	0.00
kcnn4	9.40	9.21	9.17	9.14	7.65	7.70	7.73	7.69
kcnk5	11.32	11.06	11.13	10.99	10.62	10.43	10.52	10.46
kcnk10	7.38	7.00	7.06	6.65	5.48	5.61	5.59	5.60
tnfrsf21	10.42	11.01	11.07	11.20	11.59	11.50	11.36	11.37
tnfsf10	11.92	12.43	12.10	12.27	10.95	10.82	10.95	10.95
tnfrsf23	5.63	5.82	5.72	6.05	6.12	6.13	5.90	5.81
tnfrsf11b	6.72	7.00	6.95	7.25	6.96	7.02	7.01	7.16
tnfsf13b	8.45	8.75	9.13	9.45	7.11	6.92	7.01	6.92
tnfaip8l3	9.08	9.41	9.43	9.34	4.49	4.45	4.45	4.44
tnfrsf13b	8.43	8.39	8.11	8.41	6.07	6.01	6.07	6.04
tnfaip3	10.01	9.74	10.02	9.72	8.74	8.93	9.03	8.83
tnfsf9	4.70	4.45	4.86	4.48	0.00	0.00	0.00	0.00
il15ra	8.21	8.15	8.43	8.41	6.31	6.21	6.24	6.18
stat2	10.78	10.69	11.09	10.99	8.89	8.88	8.91	8.84
nfkbia	10.93	10.61	10.75	10.67	10.22	10.26	10.31	10.18
nfkbiz	11.32	11.16	11.47	11.27	10.13	10.26	10.27	10.16
il18	10.60	10.01	10.22	9.90	11.47	11.56	11.42	11.48
il15	8.74	8.46	8.36	8.45	5.06	4.94	5.01	4.99
il34	7.59	7.40	7.20	7.34	7.12	7.26	7.22	7.28
ccl6	11.50	11.50	11.24	11.42	9.74	9.78	9.69	9.93
ccl8	5.27	5.11	5.25	5.46	8.16	8.18	8.36	8.42
ccl2	4.78	4.56	4.94	4.67	4.54	4.64	4.62	4.61
ccl25	13.74	13.72	13.57	13.52	12.63	12.54	12.53	12.57
cxcr4	7.80	7.39	7.75	7.48	8.66	8.66	8.85	8.89
ccr1	7.56	7.79	7.63	7.97	6.32	6.30	6.31	6.33
ccr2	6.82	6.88	6.62	7.08	5.24	5.28	5.29	5.27
ccr5	7.29	7.53	7.31	7.63	7.93	7.99	7.95	8.00
ccl22	6.45	7.13	6.47	6.76	0.00	0.00	0.00	0.00
gata5	11.09	11.44	11.33	11.45	11.27	11.25	11.21	11.09
tgfb1i	8.84	9.07	8.88	9.26	8.36	8.42	8.43	8.40
tgfbi	13.00	13.38	13.34	13.62	12.09	11.91	11.88	11.90
tgfbr3	8.37	8.43	8.15	8.29	6.69	6.65	6.63	6.63
il10ra	7.58	7.44	7.52	7.35	7.41	7.23	7.19	7.14
nt5e	12.16	12.29	12.57	12.69	7.49	7.60	7.61	7.86
trpm6	7.71	7.70	7.53	7.22	10.59	11.25	10.89	10.87
tmigd1	12.35	12.13	12.39	12.00	12.52	12.94	12.68	12.93
mboat1	9.09	8.35	8.87	8.48	10.72	11.01	10.91	10.93
ighv153	7.78	8.04	7.54	8.25	5.38	6.21	5.39	6.26
nos2	11.03	10.92	10.88	10.64	6.35	6.64	6.51	6.52
