# Supplementary material for: Comparative transcriptomics from intestinal cells of permissive and non-permissive hosts during Ancylostoma ceylanicum infection reveals unique signatures of protection and host specificity
Source: Parasitology. 2023 Mar 8;150(6):511–23. doi: 10.1017/S0031182023000227 (PMC10192101; doi:10.1017/S0031182023000227)
Supplement: Supplementary file 1 [file S0031182023000227sup.zip › S0031182023000227sup003.pdf]

Gene	Log fold change							
	Mouse				Hamster			
	16 hrs	24 hrs	36 hrs	adj. p value	16 hrs	24 hrs	36 hrs	adj. p value
il13ra2	1.30	1.60	3.32	**	-0.09	-0.24	0.09	ns
il4ra	0.32	0.06	0.40	*	-0.56	-0.54	-0.65	***
bcl6	0.46	0.79	0.71	***	-1.13	-0.08	-0.93	*
jun	0.22	0.36	0.48	*	-1.00	-0.74	-0.98	***
gngt1	5.53	4.99	5.65	*	0.00	0.00	0.00	ns
gng2	0.33	0.06	0.37	*	-0.05	-0.02	0.06	ns
gng11	0.66	0.45	0.66	*	0.24	0.01	0.18	ns
alox5ap	-0.38	-0.28	0.45	*	-0.12	0.17	0.05	ns
dclk1	-0.61	-0.47	-0.39	*	-0.48	-0.65	-0.33	ns
trpm5	-0.73	0.05	-0.05	*	0.22	0.22	0.60	ns
nrep	0.55	-0.04	0.66	*	0.29	-0.05	0.19	ns
sox4	0.37	0.36	0.66	***	-0.03	0.04	0.07	ns
ctsl	0.22	0.26	0.77	***	-0.45	-0.78	-0.51	***
tlr3	0.52	0.62	0.55	***	-0.06	-0.13	-0.17	ns
kcnj15	1.16	0.95	0.77	*	-0.31	-0.03	0.10	ns
kcnj13	0.99	1.11	1.96	***	0.00	0.00	0.00	ns
kcnmb1	0.60	0.35	0.74	*	-0.33	-0.17	-0.15	ns
kcne3	0.25	0.66	0.57	***	0.31	0.22	0.14	ns
kcnq1	0.23	0.29	0.49	*	-0.31	-0.24	-0.24	ns
kcnj16	-0.17	1.86	3.56	***	0.00	0.00	0.00	ns
kcnn4	-0.31	-0.36	-0.42	*	0.08	0.15	0.07	ns
kcnk5	-0.39	-0.28	-0.48	**	-0.32	-0.15	-0.26	ns
kcnk10	-0.77	-0.67	-1.63	*	0.57	0.50	0.55	ns
tnfrsf21	0.91	1.00	1.18	***	-0.13	-0.34	-0.34	ns
tnfsf10	0.73	0.27	0.51	***	-0.20	0.00	0.02	ns
tnfrsf23	0.72	0.38	1.35	*	0.22	-0.77	-1.19	ns
tnfrsf11b	0.68	0.56	1.20	***	0.18	0.15	0.51	ns
tnfsf13b	0.55	1.14	1.62	***	-0.49	-0.25	-0.48	ns
tnfaip8l3	0.53	0.56	0.42	*	-0.37	-0.37	-0.44	ns
tnfrsf13b	-0.10	-0.59	-0.06	*	-0.24	0.04	-0.09	ns
tnfaip3	-0.42	0.01	-0.45	***	0.36	0.52	0.19	ns
tnfsf9	-1.69	0.60	-1.40	*	0.00	0.00	0.00	ns
il15ra	-0.09	0.38	0.35	*	-0.31	-0.23	-0.42	ns
stat2	-0.13	0.45	0.33	*	-0.01	0.05	-0.09	ns
nfkbia	-0.48	-0.27	-0.39	*	0.07	0.18	-0.05	ns
nfkbiz	-0.24	0.21	-0.09	*	0.21	0.22	0.04	ns
il18	-0.90	-0.58	-1.07	***	0.14	-0.08	0.02	ns
il15	-0.48	-0.63	-0.48	*	-0.73	-0.30	-0.39	ns
il34	-0.38	-0.80	-0.49	*	0.37	0.26	0.41	ns
ccl6	-0.03	-0.38	-0.14	ns	0.05	-0.10	0.31	*
ccl8	-0.70	-0.02	0.71	ns	0.06	0.41	0.53	**
ccl2	-1.39	0.61	-0.64	*	0.76	0.62	0.59	ns
ccl25	-0.02	-0.24	-0.31	*	-0.14	-0.14	-0.09	ns
cxcr4	-0.80	-0.10	-0.62	*	0.01	0.40	0.41	ns
ccr1	0.46	0.14	0.78	***	-0.08	-0.02	0.02	ns
ccr2	0.11	-0.52	0.54	*	0.18	0.23	0.12	ns
ccr5	0.50	0.05	0.69	*	0.13	0.03	0.16	ns
ccl22	1.52	0.03	0.79	***	0.00	0.00	0.00	ns
gata5	0.52	0.35	0.53	**	-0.04	-0.11	-0.30	ns
tgfb1i	0.37	0.07	0.68	***	0.11	0.13	0.08	ns
tgfbi	0.56	0.50	0.90	***	-0.28	-0.32	-0.28	ns
tgfbr3	0.09	-0.39	-0.13	*	-0.11	-0.19	-0.16	ns
il10ra	-0.28	-0.10	-0.46	ns	-0.41	-0.52	-0.65	*
nt5e	0.10	0.51	0.67	ns	0.30	0.30	0.84	*
trpm6	-0.14	-0.47	-1.12	*	1.07	0.53	0.47	***
tmigd1	-0.37	0.00	-0.57	*	0.67	0.29	0.67	*
mboat1	-1.32	-0.46	-1.10	***	0.49	0.32	0.35	*
ighv153	0.47	-0.43	1.05	ns	4.56	0.15	4.53	*
nos2	-0.16	-0.22	-0.58	*	0.88	0.60	0.55	ns
