# Supplementary material for: Comparative transcriptomics from intestinal cells of permissive and non-permissive hosts during Ancylostoma ceylanicum infection reveals unique signatures of protection and host specificity
Source: Parasitology. 2023 Mar 8;150(6):511–23. doi: 10.1017/S0031182023000227 (PMC10192101; doi:10.1017/S0031182023000227)
Supplement: Supplementary file 1 [file S0031182023000227sup.zip › S0031182023000227sup004.docx]

**Fig S1.** Principal component analysis of mouse (a) and hamster (b) replicates at 0, 16, 24, and 36 hrs post-infection.

**Table S1.** Average normalized gene expression in candidate genes across time. Color scaling represents minimum (blue) versus maximum (red) values.

**Table S2**. Log fold changes in gene expression in candidate genes across time. Color scaling represents minimum (blue) versus maximum (red) values. Significance of differentially expressed genes is shown as adjusted p values (ns = non-significant (p-value>0.05); *, **, and *** = significant (p -value <0.05, 0.01, or 0,001, respectively).
