# Supplementary figures and images for: Comparative transcriptomics from intestinal cells of permissive and non-permissive hosts during Ancylostoma ceylanicum infection reveals unique signatures of protection and host specificity
Source: Parasitology. 2023 Mar 8;150(6):511–23. doi: 10.1017/S0031182023000227 (PMC10192101; doi:10.1017/S0031182023000227)

A

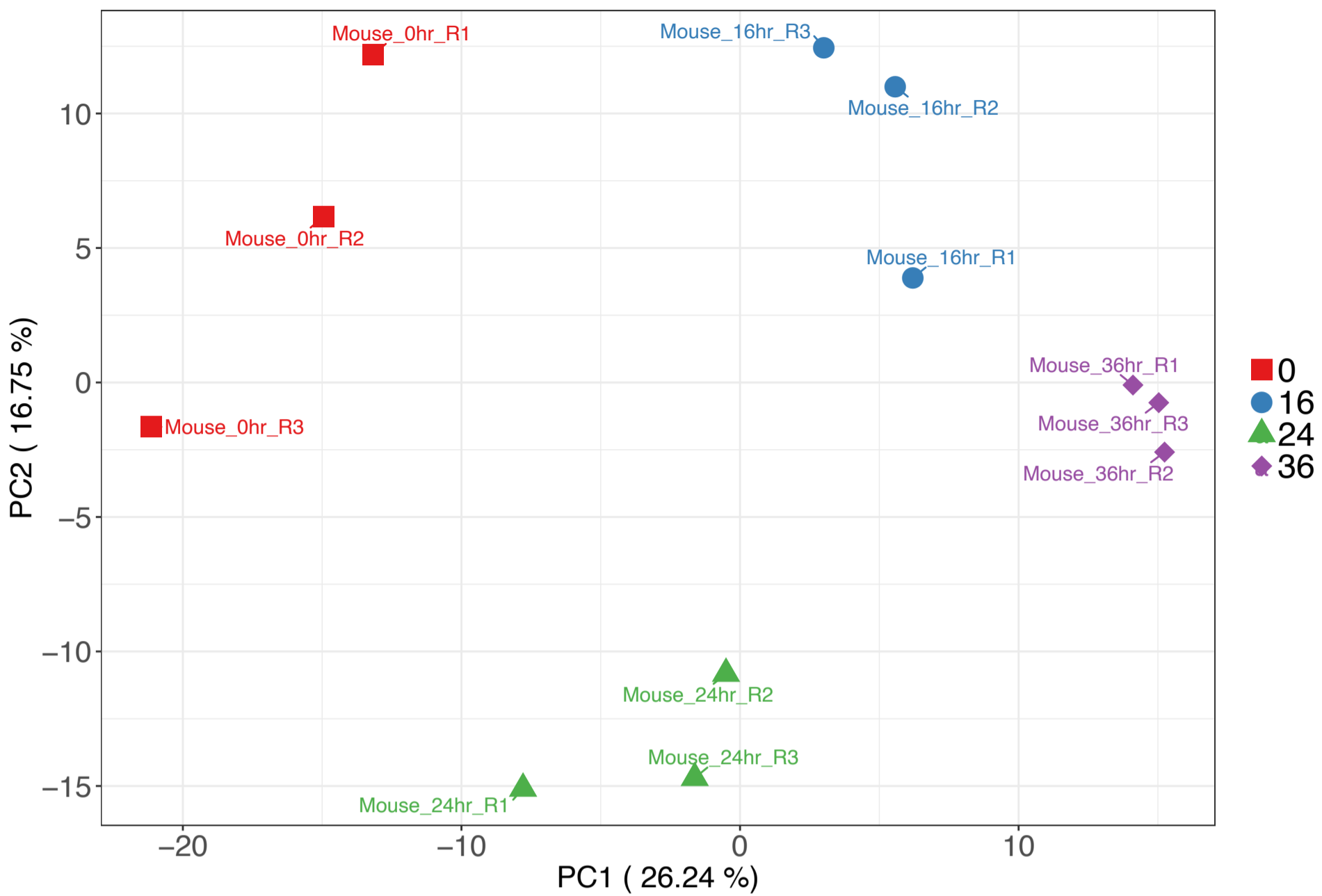

B

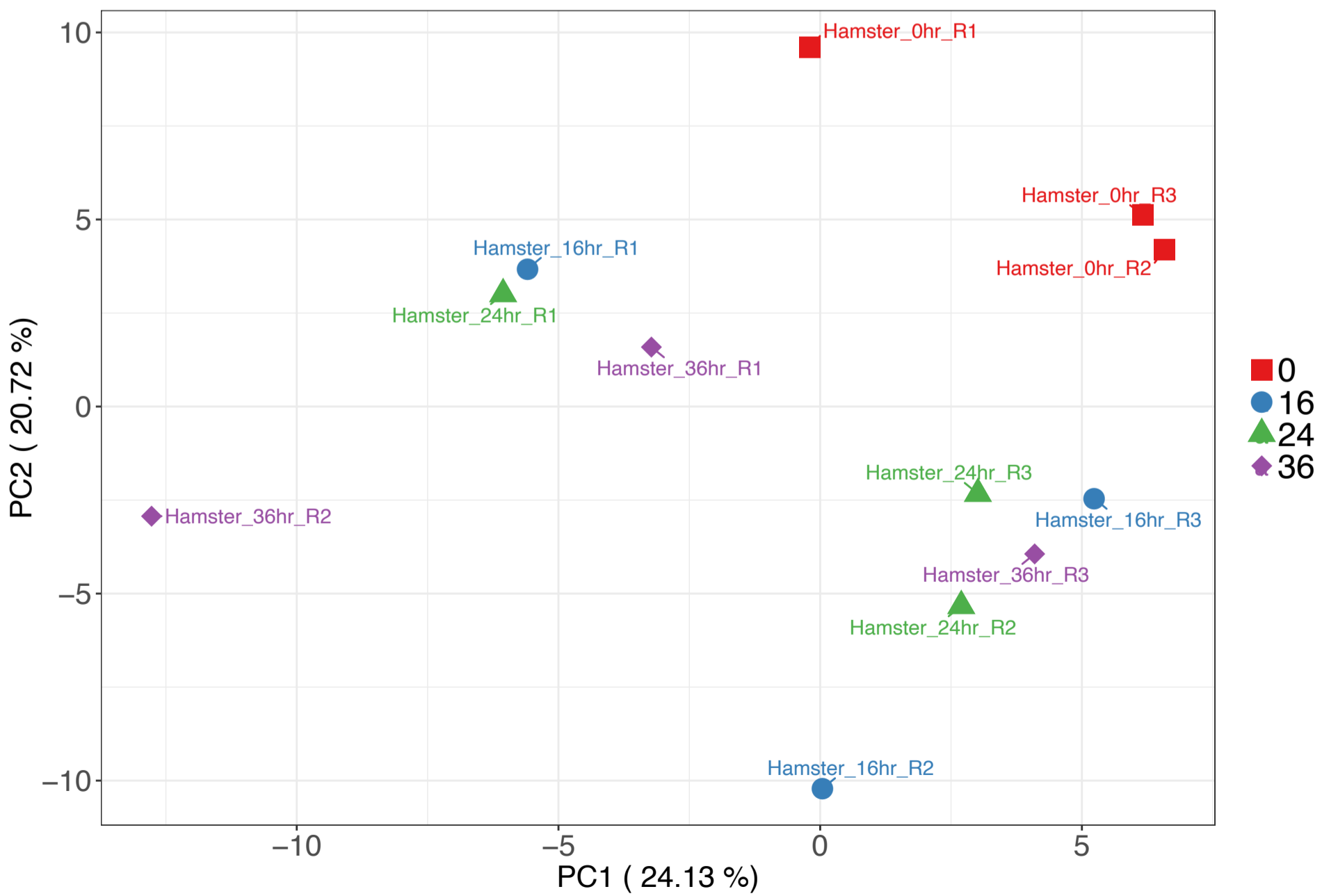

Supplement: Supplementary file 1 [file S0031182023000227sup.zip › S0031182023000227sup001.pdf]
